# Antimicrobial Susceptibility Profiles of Human *Campylobacter jejuni* Isolates and Association with Phylogenetic Lineages

**DOI:** 10.3389/fmicb.2016.00589

**Published:** 2016-04-26

**Authors:** Wonhee Cha, Rebekah Mosci, Samantha L. Wengert, Pallavi Singh, Duane W. Newton, Hossein Salimnia, Paul Lephart, Walid Khalife, Linda S. Mansfield, James T. Rudrik, Shannon D. Manning

**Affiliations:** ^1^Departments of Microbiology and Molecular Genetics, Michigan State UniversityEast Lansing, MI, USA; ^2^Department of Pathology, University of MichiganAnn Arbor, MI, USA; ^3^School of Medicine, Wayne State University, DetroitMichigan, USA; ^4^Detroit Medical Center University LaboratoriesDetroit, MI, USA; ^5^Sparrow HospitalLansing, MI, USA; ^6^Departments of Large Animal Clinical Sciences, Michigan State University, East LansingMI, USA; ^7^Bureau of Laboratories, Michigan Department of Health and Human ServicesLansing, MI, USA

**Keywords:** *Campylobacter jejuni*, antimicrobial resistance, multilocus sequence typing, epidemiology

## Abstract

*Campylobacter jejuni* is a zoonotic pathogen and the most common bacterial cause of human gastroenteritis worldwide. With the increase of antibiotic resistance to fluoroquinolones and macrolides, the drugs of choice for treatment, *C. jejuni* was recently classified as a serious antimicrobial resistant threat. Here, we characterized 94 *C. jejuni* isolates collected from patients at four Michigan hospitals in 2011 and 2012 to determine the frequency of resistance and association with phylogenetic lineages. The prevalence of resistance to fluoroquinolones (19.1%) and macrolides (2.1%) in this subset of *C. jejuni* isolates from Michigan was similar to national reports. High frequencies of fluoroquinolone-resistant *C. jejuni* isolates, however, were recovered from patients with a history of foreign travel. A high proportion of these resistant isolates were classified as multilocus sequence type (ST)-464, a fluoroquinolone-resistant lineage that recently emerged in Europe. A significantly higher prevalence of tetracycline-resistant *C. jejuni* was also found in Michigan and resistant isolates were more likely to represent ST-982, which has been previously recovered from ruminants and the environment in the U.S. Notably, patients with tetracycline-resistant *C. jejuni* infections were more likely to have contact with cattle. These outcomes prompt the need to monitor the dissemination and diversification of imported fluoroquinolone-resistant *C. jejuni* strains and to investigate the molecular epidemiology of *C. jejuni* recovered from cattle and farm environments to guide mitigation strategies.

## Introduction

*Campylobacter* spp. are Gram negative bacteria responsible for the greatest number of cases of bacterial gastroenteritis worldwide (World Health Organization [WHO], 2013). It is estimated that 1.3 million *Campylobacter* infections occur every year in the U.S., resulting in 13,000 hospitalizations and 120 deaths, with *C. jejuni* comprising almost 90% of the cases (Centers for Disease Control and Prevention [CDC], 2015). Furthermore, previous studies have demonstrated an association between campylobacteriosis and autoimmune diseases such as Guillain Barré syndrome (Nachamkin et al., 1998), reactive arthritis (Zia et al., 2003), and irritable bowel syndrome (Connor, 2005). *Campylobacter* spp. have a broad host range and can colonize the intestinal tracts of chickens, turkeys, pigs and ruminants without causing clinical signs (Altekruse et al., 1999; Stanley and Jones, 2003). These pathogens can also survive in water and soil for extended periods of time, up to several months (Jones, 2001; Pitkänen, 2013). The consumption of contaminated poultry is the primary source of sporadic human *Campylobacter* infections (Kaakoush et al., 2015), while approximately 66% of *Campylobacter* outbreaks are attributed to dairy products, mostly raw milk or cheese (Interagency Food Safety Analytics Collaboration Project, 2015). Direct transmission from animal sources including household pets such as dogs and cats, has also been reported (Damborg et al., 2004).

The most common clinical presentation of campylobacteriosis is self-limiting gastroenteritis with vomiting, cramping, and diarrhea lasting for 7–10 days in most cases (Peterson, 1994). Many individuals develop more severe and prolonged infections. Extraintestinal spread of the bacterium, for example, can lead to bacteremia and infection of other organs in some individuals (Man, 2011). Infants, geriatric patients, and immunocompromised individuals more commonly require treatment with antibiotics to combat *C. jejuni* infections (Guerrant et al., 2001). Ciprofloxacin, a fluoroquinolone that inhibits DNA synthesis by targeting *gyrA* and macrolides such as azithromycin and erythromycin, which hinder bacterial protein biosynthesis by targeting 23S rRNA, have been recommended as the first line antimicrobials. Yet, resistance to both antimicrobials has emerged and increases in resistance frequencies have been reported (Zhou et al., 2015). Indeed, the proportion of *Campylobacter* isolates resistant to fluoroquinolones has increased from 14.2% in 1998 to 25.3% in 2012 in the U.S. (Centers for Disease Control and Prevention [CDC], 2014).

*Campylobacter* resistance to fluoroquinolones and macrolides is conferred by point mutations in their target sites, the *gyrA* and 23S rRNA genes, respectively (Payot et al., 2006). The C257T point mutation in *gyrA* that yields a Thr-86-Ile amino acid change is the most frequently observed mutation that leads to high-level resistance to quinolone, i.e., nalidixic acid, and fluoroquinolone (Iovine, 2013). For macrolides, point mutations of A2074C, A2074G, and A2075G in domain V of the 23S rRNA gene, have been found to confer a high-level of resistance [Minimal inhibitory concentration (MIC) >128 μg/mL], while A2074T has been shown to confer a low-level of resistance (MIC = 8 μg/mL) (Vacher et al., 2005). The presence of the 23s rRNA methyltransferase gene *ermB* and an amino acid change in L4/L22 ribosomal proteins also have been described as resistance mechanisms for macrolides (Pérez-Boto et al., 2010; Wang et al., 2014). In addition, the active eﬄux pump, CmeABC, works synergistically with point mutations in these gene targets to simultaneously resist the action of fluoroquinolone, macrolide, tetracycline, beta-lactam, and ketolide antimicrobials (Zhang, 2008).

Tetracycline has been suggested as an alternative treatment for patients with systemic *Campylobacter* infections (Guerrant et al., 2001), but it is rarely used in practice. On the other hand, tetracycline is widely used in food animals like chickens and cattle for preventive and growth promoting purposes and treatment, e.g., in sheep for abortion (Giguere et al., 2006). In *Campylobacter*, resistance to tetracycline is conferred by *tet*(O), which has been found widely in isolates recovered from various sources (Connell et al., 2003). *tet*(O) encodes a ribosomal protection protein that induces a conformational change upon binding to the bacterial ribosome, the target site for tetracycline. This binding results in the release of the bound tetracycline molecule. A prior study has identified the *tet*(O) gene in plasmids in the majority of isolates from campylobacteriosis cases (Gibreel et al., 2004), though it has also been identified in the bacterial chromosome (Wu et al., 2014).

In the U.S., the FoodNet surveillance system was designed to monitor the incidence of common foodborne pathogens, including *Campylobacter*, while the National Antimicrobial Resistance Monitoring System (NARMS) was implemented to examine antimicrobial resistance trends. Michigan is not one of the 10 states included in the FoodNet system, however, *Campylobacter* was the most common foodborne pathogen reported through the Michigan Disease Surveillance System (MDSS) in the past decade (2004–2013). As a result, this study was undertaken to determine the frequency of antimicrobial resistance in a subset of *C. jejuni* isolates collected in Michigan between 2011 and 2012, and to estimate the genetic diversity of both susceptible and resistant isolates using multilocus sequence typing (MLST). We hypothesized that the frequency of antimicrobial resistance in *C. jejuni* isolates recovered from Michigan patients is similar to national frequencies, but that specific risk factors, which may be unique to this patient population, are associated with resistant infections. Identifying which lineages are associated with resistance and estimating their frequency in different populations is important for disease prevention efforts aimed at controlling resistance emergence and more rapidly detecting resistant infections.

## Materials and Methods

### Study Population and *Campylobacter* Isolation

From 2011 to 2012, we obtained *C. jejuni* isolates from patients with campylobacteriosis identified via the Michigan State University (MSU) Enteric Research Investigative Network (ERIN) surveillance system. This system was established in collaboration with the Michigan Department of Health and Human Services (MDHHS) and four major hospitals in Michigan. All protocols were approved by the Institutional Review Boards at Michigan State University (IRB# 10-736SM), the MDHHS (842-PHALAB), and each participating hospital.

Isolates were cultured on tryptone soy agar plates with sheep blood and cefoperazone (20 μg/mL), amphotericin B (4 μg/mL), and vancomycin (20 μg/mL) using microaerophilic conditions at 37°C for 48 h. Multiplex PCR was performed on the extracted DNA to confirm the species following previously described protocol (Yamazaki-Matsune et al., 2007). The isolates were stored in tryptone soy broth with 10% glycerol at -80°C until further testing.

### Epidemiological Data

Demographic and epidemiological data were retrieved from the MDSS^1^ and managed using Microsoft Access and Excel. Three cases were not Michigan residents, but developed campylobacteriosis while traveling in Michigan and thus, epidemiological data was transferred to the respective states. The *C. jejuni* isolates from these cases were included in the overall genetic diversity and resistance prevalence estimates, but were excluded from further analyses. A patient was considered to have a history of travel only when the traveling period was within 1 week prior to the onset of symptoms. For animal contact, birds were defined as poultry, including chickens, ducks, and turkeys, while ruminants were defined as cattle, sheep and goats, and domestic animals were defined as household pets such as dogs and cats. The season was classified based on the onset date of symptoms: spring (March, April, May), summer (June, July, August), fall (September, October, November), and winter (December, January, February). Food history represents consumption of specific foods (e.g., frozen chicken, home-prepared chicken, etc.) within a week prior to onset of symptoms.

### Phenotypic Antimicrobial Susceptibility Profiling

The MICs of nine antimicrobials were determined by a standard broth microdilution test following the guidelines of the Clinical and Laboratory Standards Institute [CLSI] (2015). The Sensititre system (Trek Diagnostic Systems, Thermo Fisher Scientific Inc., Cleveland, OH, USA) was used for each isolate following the manufacturer’s instructions. Antimicrobials included ciprofloxacin (fluoroquinolone), nalidixic acid (quinolone), azithromycin (macrolide), erythromycin (macrolide), tetracycline, florfenicol, telithromycin, clindamycin, gentamicin, and *C. jejuni* ATCC 33560 was used as the quality control strain for every batch. The breakpoints for each antimicrobial were determined using epidemiologic cut-off values (ECOFFs), following the guidelines of European Committee on Antimicrobial Susceptibility Testing [EUCAST] (2015), per current protocol of the NARMS (Centers for Disease Control and Prevention [CDC], 2014). If bacterial growth was observed at the highest MIC tested (e.g., 64 μg/mL for tetracycline), then the MIC for the isolate was interpreted as greater than the highest MIC, i.e., > 64 μg/mL.

### Whole Genome Sequencing

DNA was extracted from all 94 *C. jejuni* isolates using the Wizard genomic DNA purification kit (Promega, Madison, WI, USA) and the concentrations were measured using a Qubit fluorometer (Life Technologies; Invitrogen, Carlsbad, CA, USA). A total of 1ng of DNA per isolate was used for library preparation with the Nextera XT kit (Illumina, San Diego, CA, USA) following manufacturer’s instruction. Validation of the library size and quantity was performed using Bioanalyzer (Agilent Technologies, Santa Clara, CA, USA) and KAPA library quantification kit (Kapa Biosystems, Woburn, MA, USA), respectively. The libraries were pooled together for denaturing and sequencing on a MiSeq (Illumina) platform for 2 × 250 reads at the Research Technology Support Facility at MSU. Genomic assemblies were performed *de novo* using Velvet, 1.2.07 (Zerbino and Birney, 2008) after trimming with Trimmomatic (Bolger et al., 2014), followed by quality checking with FastQC^2^. Assemblies were constructed using different kmer values (31, 33, and 35), and the assembly yielding the best N50 value for each isolate was used for downstream analyses.

### Multilocus Sequence Typing

The MLST profile of each sample was initially determined using the web-based server^3^ with both the assembled contigs and extracted alleles from whole genome sequences. Each gene sequence was also confirmed by PCR-based MLST, as described previously (Dingle et al., 2001). Allele, sequence type (ST), and clonal complex (CC) assignments were made using the PubMLST database^4^ (Jolley and Maiden, 2010). New alleles (*n* = 4) and STs (*n* = 6) identified in this study were deposited in the PubMLST database (id 24892-28175).

### *In Silico* Analysis of Resistance Genes

Sequences specific for *gyrA* and 23S rRNA were extracted from the draft genomes based on reference sequences available via the National Center for Biotechnology Information using the Basic Local Alignment Search Tool (BLAST) (Altschul et al., 1990) (*gyrA*: GenBank KU693342-KU693435; 23S rRNA: GenBank KU891692-KU891785). Regions of the 23S rRNA and gyrA genes, which include the typical point mutation sites associated with resistance to macrolides and fluoroquinolones, respectively, were aligned by MegAlign (DNAstar, Madison, WI, USA). Sanger sequencing was used to confirm the point mutations identified in 47 strains relative to the ATCC 33560 *C. jejuni* strain (*gyrA*: GenBank KU693436; 23S rRNA KU891786), as described in prior studies (Vacher et al., 2003; Said et al., 2010). The presence of *tet*(O) was determined from the genome sequences and further confirmed by PCR using a published protocol (Gibreel et al., 2004).

### Data Analysis

The frequency map of all campylobacteriosis cases reported in Michigan between 2011 and 2012 (*n* = 1,449) was generated using ArcMap GIS software (version 10.2; ESRI, Redlands, CA, USA) using the data extracted from MDSS.

To identify evolutionary relationships between *C. jejuni* isolates, a Neighbor-joining phylogeny (p-distance) with 1,000 bootstrap replications was constructed in MEGA6 (Tamura et al., 2013) based on seven MLST loci. Clusters or CCs were classified as STs that grouped together with >70% bootstrap support, and parsimonious informative sites were further evaluated for evidence of genetic recombination using Splitstree4 (Huson and Bryant, 2006).

Statistical analyses were performed using SAS version 9.3 (SAS Institute, Cary, NC, USA). Differences in the frequencies of antimicrobial resistance across ST, CC, and other variables including disease presentation, were examined using χ^2^ and Fisher’s exact tests for dichotomous variables, and the student’s *t*-test for continuous variables; *p* < 0.05 was considered significant. Multivariate analyses were performed using logistic regression with any independent variables that yielded a *p*-value of less than 0.2 or were plausibly linked to resistant *C. jejuni* infections, e.g., age, sex. The model was built using a forward stepwise method with the requirement for a significance level of ≤0.1 to remain in the model.

## Results

### Description of *Campylobacter* Cases Identified in Michigan

Ninety four *C. jejuni* isolates were recovered from clinical cases of campylobacteriosis identified at four hospitals between January 2011 and December 2012. Among the 94 cases, more than 50% (*n* = 52) were male, while 39 cases were female patients; the sex was not known for three cases. Children 2 years and younger (22.3%; *n* = 21) and adults older than 50 years (25.5%; *n* = 24) comprised about half of the total cases. Race was available for 80 of the 94 cases, and the majority identified as Caucasian (*n* = 60; 75.0%).

**FIGURE 1:**
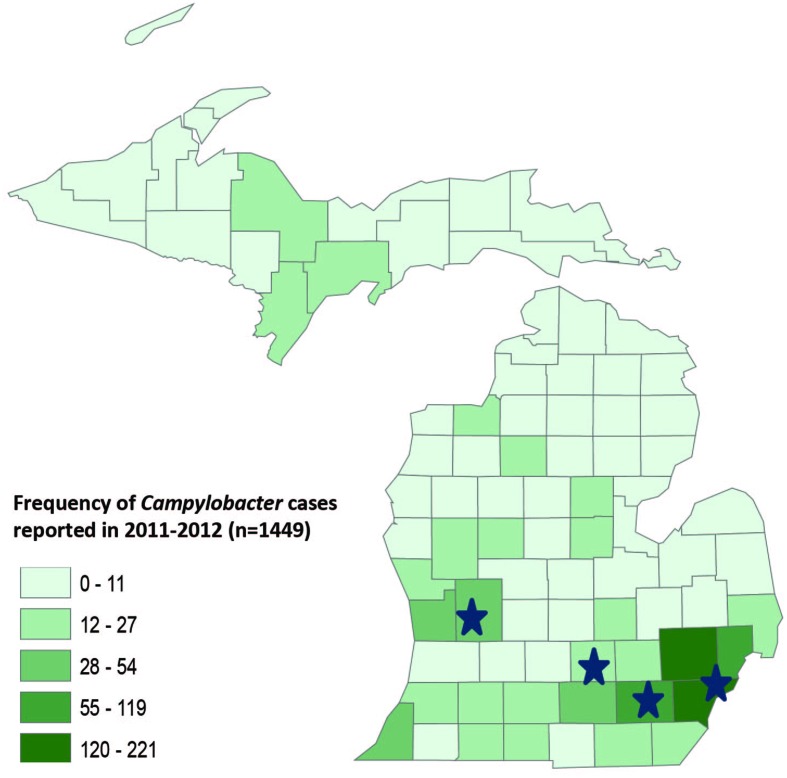
**GIS map of Michigan by county showing the frequency of *Campylobacter* cases reported in 2011–2012.** The stars represent the location of four hospitals where the samples were collected.

A total of 1,449 laboratory confirmed *Campylobacter* cases were reported in Michigan in 2011 and 2012 and the frequency of reported cases varied across counties (**Figure 1**). The participating hospitals and most of the residences of the cases included in this study were in counties with higher population densities and a greater frequency of campylobacteriosis. Particularly, cases residing in Wayne, Washtenaw and Oakland counties, which represent the counties with the highest disease frequency, comprised 66.7% (*n* = 58) of the total cases.

Sixty-eight cases had travel history information, among which nine (13.2%) had a history of foreign travel, while 13 (19.1%) had a history of domestic travel in the week prior to the onset of symptoms (**Table 1**). Four cases had a history of domestic travel outside Michigan, while three cases from other states (Ohio, New Jersey, and Georgia) developed symptoms and were diagnosed with campylobacteriosis while traveling in Michigan. Among the 64 cases with a history of animal contact, 38 (59.4%) had contact with domestic animals including dogs and cats. Contact with ruminants, which were all identified as cattle, was reported in seven cases (10.9%), while six cases (9.4%) reported contact with birds such as chickens and ducks. Six of the cases reporting contact with cattle (85.7%) and five of the cases with bird contact (83.3%) also reported contact with dogs and cats.

**Table 1:** Description of cases included in the study.

Demographic data	No. of cases^†^	(%)^‡^	Epidemiologic data	No. of cases^†^	(%)^‡^
**Sex**			**Travel**		
Male	52	57.1%	No travel	46	67.6%
Female	39	42.9%	Domestic travel	13	19.1%
**Age group (years)**			Foreign travel	9	13.2%
≤2	21	22.3%	**Animal contact**		
3–23	25	26.6%	Reptile	0	0%
24–50	24	25.5%	Livestock	7	10.9%
>50	24	25.5%	Birds/poultry	6	9.4%
Race			Domestic	38	59.4%
**Caucasian**	60	75.0%	Others	5	7.8%
African American	9	11.3%	**Food consumption**		
Asian	1	1.2%	Ground meats	33	55.0%
Others	10	12.5%	Home prepared chickens	30	50.0%
**Residence (county)**			Frozen chickens	14	23.3%
Clinton	4	4.6%	Restaurant chickens	19	31.7%
Ingham	8	9.2%	Raw sprouts	4	6.7%
Livingston	6	6.9%	Raw milk	4	6.7%
Macomb	3	3.4%	**Water at home**		
Oakland	10	11.5%	Well	12	19.0%
Washtenaw	17	19.5%	Municipal	42	66.7%
Wayne	31	35.6%	Bottled	7	11.1%
Others	8	9.2%	Others	2	3.2%

*^†^ Counts for sex, age group, race, travel history, and water source at home are mutually exclusive for each category; counts for animal contact and food consumption are repeated across categories as they are reported. ^‡^ The percentages are based on the number of cases for which information was available. Total number of cases varies between variables due to the difference in missing information.*

### Antimicrobial Resistance Profiles of *C. jejuni* Isolates and Mechanisms of Resistance

Thirty isolates (31.9%) were susceptible to all nine antimicrobial agents tested, while 64 isolates (68.1%) were resistant to one or more agents. The highest frequency of resistance was observed for tetracycline (*n* = 58 isolates; 61.7%), followed by resistance to both ciprofloxacin and nalidixic acid (*n* = 18 isolates; 19.1%) (**Table 2**). All *C. jejuni* isolates resistant to ciprofloxacin and nalidixic acid showed high MICs (4–32 μg/mL, ≥64 μg/mL, respectively). Resistance to florfenicol was detected in one isolate (1.1%), and all isolates were susceptible to gentamicin. A total of 15 isolates (16.0%) were resistant to two or more classes of antibiotics. Thirteen (13.8%) of these isolates were resistant to ciprofloxacin, nalidixic acid, and tetracycline, while two isolates (2.1%) were resistant to both azithromycin and erythromycin as well as ciprofloxacin, nalidixic acid, telithromycin, and clindamycin.

**Table 2:** Frequency (%) of antimicrobial resistance and minimum inhibitory concentration (MIC) of human *C. jejuni* isolates in Michigan.

CLSI Antimicrobial class	Antimicrobial agent	% Resistance (*n* = 94)	MIC (μg/mL)		
			Range	MIC_50_	MIC _90_
Fluoroquinolone	Ciprofloxacin	19.15% (18)	<0.015 ∼ 32	0.12	8
Quinolone	Nalidixic acid	19.15% (18)	<4 ∼ > 64	4	>64
Macrolide	Azithromycin	2.13% (2)	<0.015 ∼ > 64	0.06	0.12
	Erythromycin	2.13% (2)	< 0.03 ∼>64	0.5	1
Aminoglycoside	Gentamicin	0	< 0.12 ∼ 1	0.5	1
Tetracycline	Tetracycline	61.7% (58)	< 0.06 ∼ > 64	64	>64
Lincosamide	Clindamycin	2.13% (2)	< 0.03 ∼ > 16	0.12	0.25
Ketolide	Telithromycin	2.13% (2)	<0.015 ∼ > 8	1	2
Phenicol	Florfenicol	1.06% (1)	< 0.03 ∼ 32	1	2

*^†^ CLSI: Clinical and Laboratory Standards Institute.*

All 18 *C. jejuni* isolates that were phenotypically resistant to ciprofloxacin had a point mutation at 257 in *gyrA*; 17 isolates had the C257T mutation resulting in an amino acid change of Thr-86-Ile, while one isolate had double mutations (C257G and A258G) resulting in a Thr-86-Arg change. Two isolates that were resistant to azithromycin and erythromycin had an A2074T point mutation in 23S rRNA, and all 58 tetracycline resistant isolates harbored *tet*(O).

### Epidemiological Associations with Antimicrobial Resistant *C. jejuni* Infections

To identify factors associated with antimicrobial resistant *C. jejuni* infections, we conducted univariate analyses using demographic and epidemiological data (Supplementary Table S1). Notably, cases reporting a history of foreign travel had a higher likelihood of fluoroquinolone-resistant *C. jejuni* infections (Fisher’s *p* < 0.0001) with an odds ratio (OR) of 35.7 (exact 95% CI; 4.6, 395.3). In detail, among the nine cases with foreign travel history, seven were resistant to ciprofloxacin and nalidixic acid; six of these isolates were also resistant to tetracycline, yielding a significant association between foreign travel and the following resistance profile: ciprofloxacin-, nalidixic acid-, tetracycline-resistance (CipNalTet) (Fisher’s *p* < 0.0001; OR = 35.3). Cases with CipNalTet infections were significantly more common during the winter months of December, January, and February (Fisher’s *p* < 0.05; OR = 5.7), while eating chicken prepared at home was protective for both CipNalTet infections (*p* < 0.01; OR = 0.0) and fluroquinolone-resistant infections (*p* < 0.01; OR = 0.08). By contrast, contact with cattle was associated with tetracycline-resistant infections (Fisher’s *p* < 0.05; OR = infinity) and cases reporting consumption of frozen chicken were less likely to have tetracycline-resistant infections (*p* < 0.05; OR = 0.2).

Multivariate analysis was conducted to identify predictors of fluoroquinolone-resistant infections in all 94 cases using the factors with significant associations (*p* < 0.2) identified in the univariate analyses as well as biologically plausible factors such as age, sex (**Table 3**). History of foreign travel was associated with fluoroquinolone-resistant infections regardless of age and sex; the OR was 33.4 (95% CI = 3.9–285.2) in the final model. When the same multivariate analysis was performed for CipNalTet infections, history of foreign travel (OR = 54.2; 95% CI = 4.1–717.1) and winter (OR = 25.3; 95% CI = 1.6–405.7) were independently associated with infection. However, there was no association between foreign travel history and season, including winter. Although having chicken prepared at home was a protective factor in the univariate analysis, this variable could not be examined in the multivariate models as it significantly reduced the sample size due to missing data in 37 of the cases.

**Table 3:** Univariate and multivariate analyses of factors associated with fluoroquinolone resistant *C. jejuni* infections among all cases (*n* = 94).

Characteristic	Univariate analysis			Multivariate analysis		
	OR	95% CI^†^	*p*	OR	95% CI^†^	*p*
Foreign travel	35.7	5.78–220.38	<0.0001	33.4	3.9–285.2	0.0013
Season (Winter)	3.27	0.92–11.58	0.056	8.1	0.9–72.7	0.0614
Age (years)*	–	–	–	1.05	0.99–1.1	0.0536
Sex (Female)	0.92	0.32–2.68	0.88	–	–	–
Domestic animal contact	0.37	0.10–1.33	0.19	0.26	0.041–1.659	0.1542
Home prepared chicken**	0.082	0.0095–0.71	0.0095	–	–	–

*Age (years) is a continuous variable; not proper for univariate analysis used (χ^2^ or Fisher’s exact test). **Consumption of home prepared chicken was a significant protective factor by univariate analysis; however, it was not used for multivariate analysis because of the small sample size left when the characteristic was included in the base model. ^†^Wald Confidence interval

### Genetic Diversity and Phylogenetic Structure of *C. jejuni*

**FIGURE 2:**
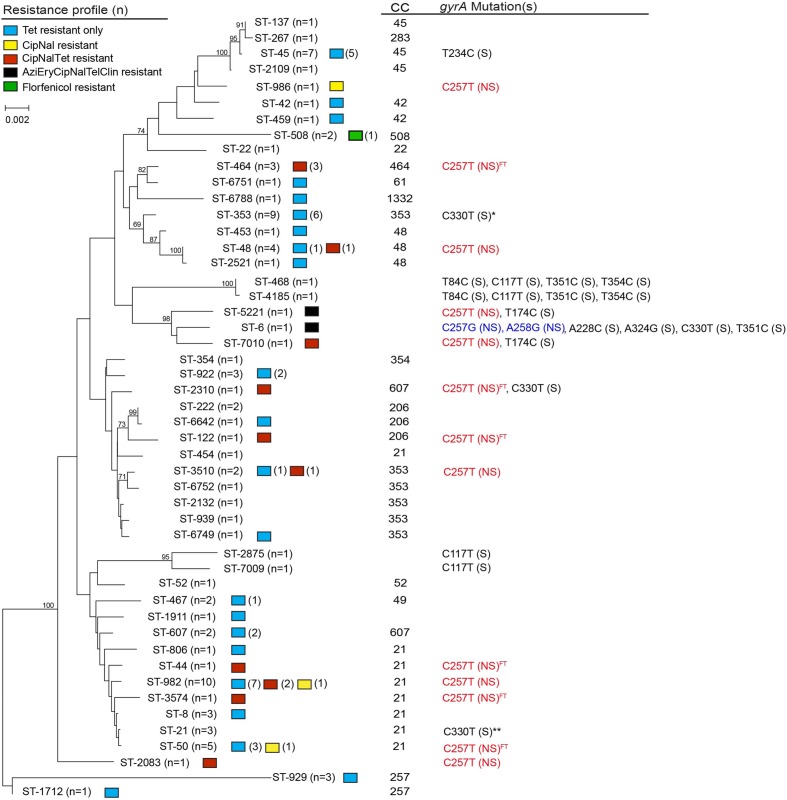
**Neighbor-joining phylogeny of sequence types (STs) by antimicrobial resistance patterns, clonal complexes (CCs), and type of *gyrA* mutation.** The numbers at the end of each branch represent the STs found in this study, while the numbers at the nodes represent the bootstrap value from 1,000 replications. Non-synonymous mutation is noted by NS in parentheses; S in parentheses indicates synonymous mutation. Superscript FT indicates isolates recovered from a case with foreign travel history. *gyrA* mutations were determined using *C. jejuni* ATCC 33560 as the reference; location 243, 357, 360 were defined as CT variable regions. *6 of 9 isolates had a synonymous point mutation at C330T. **1 of 3 isolates had a synonymous point mutation at C330T.

A total of 49 different STs, including six novel STs, were represented among the 94 *C. jejuni* isolates recovered in Michigan (**Figure 2**). These STs were assigned to 17 CCs, while 11 STs were singletons. The six new STs were assigned to ST-6749 (CC-353), ST- 6751 (CC-61), ST-6752 (CC-353), ST-6788 (CC-1332), ST-7009 (CC unassigned), and ST-7010 (CC unassigned). The most prevalent STs were ST-982 (*n* = 10; 10.6%) and ST-353 (*n* = 9; 9.6%), followed by ST-45 (*n* = 7; 7.4%), ST-50 (*n* = 5; 5.3%) and ST-48 (*n* = 4; 4.3%). Thirty four of the remaining STs had only one isolate assigned to each ST.

**FIGURE 3:**
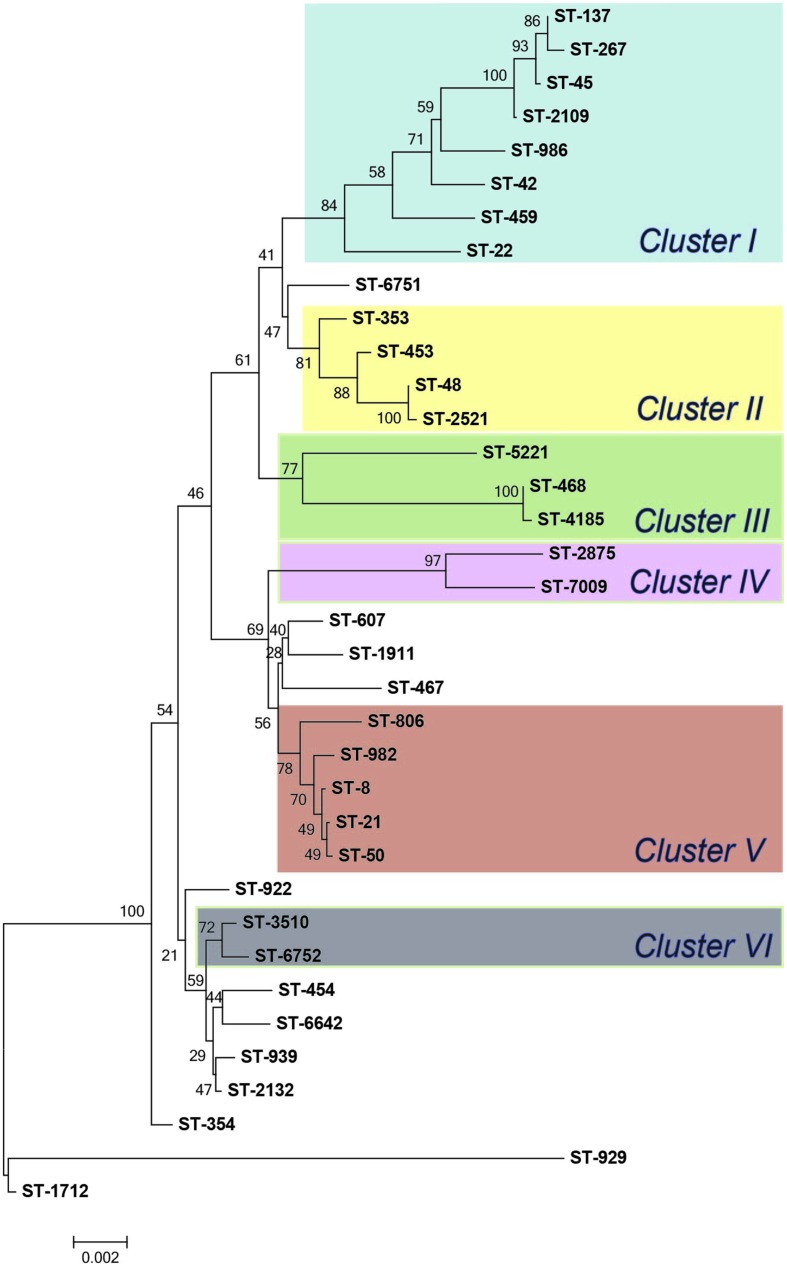
**Neighbor-joining phylogeny of sequence types (STs) from Michigan (*n* = 52).** The numbers at the end of each branch represent the STs and numbers at the nodes are bootstrap values following 1,000 replications.

The MLST-based Neighbor-joining phylogeny for all 94 isolates showed that some STs were closely related, though the bootstrap values were low, which is likely due to the high diversity and frequent recombination among STs in this isolate population. Indeed, an evaluation of the 144 parsimonious informative sites provided evidence of significant recombination [pairwise homoplasy index (PHI) = 0.0] among the STs via a Neighbor-net analysis (Supplementary Figure S1). In order to elucidate the ST distribution and evolutionary relationships of isolates that were restrictively derived from Michigan, we excluded cases with foreign travel and history of travel outside of Michigan as well as cases with missing data. A total of 36 different STs, including four novel STs, represented the 52 Michigan cases without any history of travel (*n* = 46) or travel only within Michigan (*n* = 6). Although there was still evidence of recombination between these isolates, the phylogenetic tree showed enhanced bootstrap support, and six distinct clusters with bootstrap support values exceeding 70% were identified (**Figure 3**, Supplementary Figure S2).

### Association between Phylogenetic Lineage, Epidemiologic Data, and Resistance

Multiple epidemiological factors were associated with specific *C. jejuni* genotypes. For example, individuals reporting a history of foreign travel were more likely to have infections caused by ST-464 (CC-464) isolates (Fisher’s *p* < 0.05), while infection with ST-982 was associated with cattle contact (Fisher’s *p* < 0.05). Drinking water obtained from a well at home (Fisher’s *p* < 0.05), and contact with birds, i.e., chickens (Fisher’s *p* < 0.01) were also associated with ST-982 infections, while contact with birds, i.e., chickens (Fisher’s *p* < 0.05) and female gender (χ^2^ *p* < 0.05) were both associated with CC-21 (*n* = 25) infections. In addition, cases older than 50 years of age were more likely to have isolates belonging to CC-45 (*n* = 9) relative to all other CCs (Fisher’s *p* < 0.05).

Among the 52 cases without any history of travel or travel only within Michigan, similar associations were observed. Notably, contact with birds, i.e., chickens, was associated with both ST-982 (Fisher’s *p* < 0.01) and CC-21 (Fisher’s *p* < 0.05) infections. Because the phylogenetic clusters were better defined in the isolate population recovered exclusively from Michigan cases (**Figure 3**), we also analyzed epidemiologic associations by clusters identified in the Neighbor joining phylogeny (**Figure 3**). Isolates belonging to Cluster V, which includes ST-982 and the 4 other STs, all representing CC-21, were significantly associated with contact with birds, i.e., chickens (Fisher’s *p* < 0.05). Additionally, when compared to other clusters, the two cases reporting contact with cattle were included in Cluster V; however, the association was not significant (Fisher’s *p* = 0.0571), which may be due to the small sample size.

It is also notable that the three isolates assigned to ST-464 had the same profile with resistance to ciprofloxacin, nalidixic acid and tetracycline (CipNalTet). A significant association was observed between ST-464 and resistance to ciprofloxacin and nalidixic acid (Fisher’s *p* < 0.01), as well as CipNalTet (Fisher’s *p* < 0.01). Similarly, the isolates belonging to ST-982 had a higher likelihood of having resistance to tetracycline when compared to all other lineages (Fisher’s *p* < 0.05). No other significant associations were observed for other STs or CCs with specific resistance profiles.

## Discussion

Increasing frequencies of resistance to antimicrobials in *C. jejuni* is a growing public health concern worldwide. In the U.S., NARMS monitors resistance trends by testing *C. jejuni* isolates collected from 10 states across the country; however, Michigan is not included in this system. Hence, this represents the first study reporting the antimicrobial resistance profiles and genetic diversity of *C. jejuni* recovered from patients in Michigan collected via active surveillance. The high frequency of resistance to several important antimicrobials including the fluoroquinolones and macrolides, which are commonly used to treat human infections, is of concern as are the risk factors for resistant infections. Indeed, we found that a history of foreign travel is the most important risk factor for fluoroquinolone-resistant *C. jejuni* infections in Michigan, which is consistent with prior reports in other geographic locations in the U.S. (Smith et al., 1999; Kassenborg et al., 2004) and Europe (Engberg et al., 2001; Rautelin et al., 2003; Norström et al., 2006). A case-control study conducted using FoodNet surveillance sites in the U.S. during 1998–1999, for instance, demonstrated that history of foreign travel resulted in a higher likelihood of acquiring fluoroquinolone-resistant *Campylobacter* infections (OR = 7.6; 95% CI = 4.3-13.4) (Kassenborg et al., 2004). This same study also found that consumption of poultry prepared outside of the home was a risk factor for domestically acquired fluoroquinolone-resistant infections (OR = 10.0, 95% CI = 1.3–78). The latter result is similar to our finding that chicken prepared at home was protective and resulted in a decreased likelihood of having a fluoroquinolone-resistant infection. These data are important as they demonstrate that the major sources of fluoroquinolone-resistant *C. jejuni* infections are similar in different parts of the U.S. even though disease frequencies may vary. They also emphasize the importance of monitoring patients with foreign travel history and considering individual treatment schemes in order to more readily combat resistant infections.

Prior studies have suggested that a high frequency of travel-associated fluoroquinolone-resistant *Campylobacter* infections may be due to a high prevalence of fluoroquinolone resistance in the destination areas (Piddock, 1995; Smith et al., 1999). Recently, clonal spread of specific fluoroquinolone-resistant lineages, e.g., ST-464, have been reported in Europe (Cody et al., 2012; Kittl et al., 2013). According to the PubMLST database (Jolley and Maiden, 2010), ST-464 has been frequently reported in Europe and Asia, however, no prior reports have been noted in the U.S. All three isolates assigned to ST-464 had the same resistance profile (CipNalTet) and two of these isolates were from cases that had traveled to Cambodia and to Italy, Turkey, Greece, and France; travel status was missing for the remaining case. These data suggest the dissemination of a fluoroquinolone-resistant ST-464 lineage outside of Europe, warranting the need to enhance surveillance efforts and identify factors associated with global spread.

From a total of 94 isolates, thirty six (38.3%) had at least one point mutation in *gyrA* compared to the ATCC 33560 reference strain that is susceptible to all antimicrobials tested (McDermott et al., 2004). Eighteen isolates had non-synonymous point mutations conferring resistance to fluoroquinolones; the amino acid changes, Thr-86-Ile and Thr-86-Arg, were observed in 17 isolates and 1 isolate, respectively. Although the Thr-86-Arg mutation was described as one of the mutations inducing high MIC values for fluoroquinolones in *Campylobacter* spp. (Rossi et al., 2015), this mutation is rarely reported. It is also notable that only this isolate with Thr-86-Arg had transversion mutations, C257G and A228C in *gyrA* (**Figure 2**). Interestingly, among the 17 isolates with a Thr-86-Ile mutation, 13 different STs were represented and five of these were recovered from patients with a history of foreign travel to multiple geographic locations. These data demonstrate that fluoroquinolone resistance can develop via similar point mutations in *gyrA* among phylogenetically distinct *Campylobacter* lineages. No other pattern or association was observed in the type or frequency of synonymous point mutations in *gyrA* and specific resistance profiles or increased MICs. However, notable associations were observed between STs and the types of point mutations. For example, all seven isolates assigned to ST-45 had a T234C point mutation, while the two closely related lineages, ST-2875 and ST-7009, ST-468 and ST-4185, respectively, contained identical synonymous *gyrA* point mutations (**Figure 2**). Indeed, prior studies have suggested the use of *gyrA* as a genetic marker to investigate the relatedness of *C. jejuni* strains (Ragimbeau et al., 2014). It is also important to note that not all mutations were restricted to closely related lineages. The C330T synonymous mutation in *gyrA*, for instance, was found in ST-353, ST-6, ST-2310, and ST-21, distinct lineages located in different branches of the phylogenetic tree.

Prior studies have detected variation in the level of resistance to macrolides based on the type of mutation in 23S rRNA as well. The A2074T mutation in 23S rRNA, for example, has been linked to low macrolide resistance levels previously (Vacher et al., 2005), while a recent study detected a high resistance level (MIC > 512 μg/ml) in a *C. jejuni* strain from a human clinical case (Ohno et al., 2016). Similar to the latter finding, the two macrolide resistant isolates recovered from Michigan had the same A2074T 23S rRNA mutation with high resistance levels to both erythromycin and azithromycin (MIC > 64 μg/ml). Notably, these isolates were multiple drug resistant (MDR) with resistance to four classes of antimicrobials including macrolides, a fluoroquinolone, a lincosamide (clindamycin) and a ketolide (telithromycin), suggesting that other resistance mechanisms could impact the overall level of resistance. One example is the *cmeABC* eﬄux pump, which has been linked to resistance to multiple antimicrobials, including fluoroquinolones, macrolides, and tetracycline. Both of the Michigan strains, however, were susceptible to tetracycline. It is important to note that these isolates, representing ST-5221 and ST-6, respectively, clustered together in the Neighbor-joining phylogeny with ST-7010 (**Figure 2**), all of which contained a high frequency of similar synonymous point mutations in 23S rRNA (Supplementary Table S2). This finding suggests that some *C. jejuni* lineages may be more susceptible to point mutations, which may lead to antimicrobial resistance. Further studies are required to determine whether specific synonymous mutations impact the mutability of some isolates resulting in greater mutation rates or enhanced fitness *in vivo*.

A significantly higher tetracycline resistance rate (61.7%) was observed in the study samples when compared to the 2012 NARMS report (47.8%) (χ^2^ *p* < 0.01). All of the tetracycline-resistant *C. jejuni* isolates (*n* = 58) harbored *tet*(O), which was confirmed by both PCR and *in silico* analysis. Although we could not determine whether *tet*(O) was plasmid-derived in all of the isolates examined, the gene was found in multiple lineages throughout the phylogeny, suggesting that plasmid-mediated resistance and horizontal gene transfer across lineages is likely. In addition, a significant association was observed between ST-982 and tetracycline resistance as well as cattle contact. According to the PubMLST database, ST-982 has previously been reported from cattle (*n* = 14), cow’s milk (*n* = 4), the farm environment (*n* = 3) and a lamb (*n* = 1) in the U.S., while it was linked to human clinical cases in Canada and the U.K. Another study conducted in the state of Washington (Davis et al., 2013), however, recovered ST-982 isolates from both humans and cattle. Taken together, these data suggest that this resistant genotype has the ability to cross species and may be more likely to acquire resistance to tetracycline. A significant association was also observed between ST-982 and contact with chickens and drinking well water at home, suggesting that this lineage may be readily adaptable to different hosts and environments. Further investigation of the genetic diversity and antimicrobial resistance profiles of *C. jejuni* recovered from reservoir hosts and the environment, however, is needed to better understand transmission dynamics and host specificity.

Recent studies in Europe show mounting evidence that clonal spread of specific fluoroquinolone-resistant *C. jejuni* lineages may be contributing to the increasing trend of fluoroquinolone resistance observed worldwide (Cody et al., 2012; Wimalarathna et al., 2013; Kovač et al., 2014). Our study affirms foreign travel as a major risk factor for fluoroquinolone-resistant infections in the U.S., and further suggests that specific resistant lineages, like ST-464, may be emerging in the country. With continuing globalization through imported food and foreign travel, unified diagnostic guidelines on resistance detection and global surveillance is warranted to track the emergence and spread of these resistant lineages so effective mitigation strategies can be implemented.

## Author Contributions

WC, JR, SM designed the study; WC, RM, SW, PS, SM generated data; WC, DN, HS, PL, WK, LM, JR, and SM analyzed the data; and WC, SM drafted the manuscript. All authors read and approved the final manuscript.

## Conflict of Interest Statement

The authors declare that the research was conducted in the absence of any commercial or financial relationships that could be construed as a potential conflict of interest.

## Footnotes

1 http://www.michigan.gov/mdhhs/0,5885,7-339-71550_5104_31274---,00.html

2 http://www.bioinformatics.babraham.ac.uk/projects/fastqc/

3 http://www.cbs.dtu.dk/services/MLST

4 http://pubmlst.org/campylobacter/
