# Supplementary material for: Antimicrobial Susceptibility Profiles of Human Campylobacter jejuni Isolates and Association with Phylogenetic Lineages
Source: Front Microbiol. 2016 Apr 26;7:589. doi: 10.3389/fmicb.2016.00589 (PMC4845714; doi:10.3389/fmicb.2016.00589)
Supplement: Supplementary file 1 [file Table_1.DOCX]

**Supplementary Table 1.** Univariate analyses of potential factors associated with antimicrobial resistant *C. jejuni* infections among all cases (*n*=94) and cases from Michigan only (*n*=52).

| **Characteristic** | **All cases (*n*=94)** | | | | | | | **Michigan cases (*n*=52)** | | | | | | |
| --- | --- | --- | --- | --- | --- | --- | --- | --- | --- | --- | --- | --- | --- | --- |
|  | **Total No** | **FQ-res^α^** | ***p^δ^*** | **TET-res^β^** | ***p^δ^*** | **FQTET-res^γ^** | ***p^δ^*** | **Total No** | **FQ-res^α^** | ***p^δ^*** | **TET-res^β^** | ***p^δ^*** | **FQTET-res^γ^** | ***p^δ^*** |
| **Age** |  |  |  |  |  |  |  |  |  |  |  |  |  |  |
| **≤4** | 26 | 4 | .85 | 18 | .4 | 3 | .93 | 12 | 0 | .67 | 10 | .06 | 0 | 1.0 |
| **5~49** | 39 | 8 |  | 21 |  | 6 |  | 22 | 2 |  | 9 |  | 1 |  |
| **≥50** | 29 | 6 |  | 19 |  | 4 |  | 18 | 2 |  | 10 |  | 1 |  |
| **Sex** |  |  |  |  |  |  |  |  |  |  |  |  |  |  |
| **Male** | 52 | 10 | .88 | 29 | .19 | 6 | .59 | 28 | 3 | .62 | 14 | .27 | 1 | 1.0 |
| **Female** | 39 | 7 |  | 27 |  | 6 |  | 23 | 1 |  | 15 |  | 1 |  |
| **Race** |  |  |  |  |  |  |  |  |  |  |  |  |  |  |
| **Caucasian** | 60 | 12 | .74 | 36 | .93 | 10 | 1.0 | 38 | 3 | .46 | 19 | 1.0 | 2 | 1.0 |
| **Non-caucasian** | 17 | 4 |  | 10 |  | 2 |  | 6 | 1 |  | 3 |  | 0 |  |
| **Residence** |  |  |  |  |  |  |  |  |  |  |  |  |  |  |
| **Wayne** | 30 | 6 | .55 | 19 | .58 | 4 | .21 | 13 | 0 | .39 | 10 | .07 | 0 | .07 |
| **Washtenaw** | 17 | 5 |  | 11 |  | 5 |  | 12 | 2 |  | 7 |  | 2 |  |
| **Oakland** | 10 | 1 |  | 4 |  | 1 |  | 7 | 0 |  | 1 |  | 0 |  |
| **Other** | 30 | 4 |  | 18 |  | 2 |  | 20 | 2 |  | 11 |  | 0 |  |
| **Travel** |  |  |  |  |  |  |  |  |  |  |  |  |  |  |
| **Foreign travel** | 9 | 7 | <.0001 | 7 | .52 | 6 | <.0001 | - | - |  | - |  | - |  |
| **Domestic travel** | 13 | 1 |  | 8 |  | 1 |  | 5 | 0 | 1.0 | 2 | .64 | 0 | 1.0 |
| **No travel** | 46 | 4 |  | 27 |  | 2 |  | 47 | 4 |  | 27 |  | 2 |  |
| **Animal contact†** |  |  |  |  |  |  |  |  |  |  |  |  |  |  |
| **Domestic animal** | 38 | 5 | .19 | 22 | .49 | 3 | .077 | 32 | 3 | 1.0 | 17 | .70 | 1 | 1.0 |
| **Livestock** | 7 | 0 | .33 | 7 | .037 | 0 | .58 | 5 | 0 | 1.0 | 5 | .054 | 0 | 1.0 |
| **Birds/poultry** | 6 | 0 | .58 | 5 | .39 | 0 | .58 | 6 | 0 | 1.0 | 5 | .20 | 0 | 1.0 |
| Supplementary Table1. (cont’d) | | | | | | | | | | | | | | |
| **Food consumption†** |  |  |  |  |  |  |  |  |  |  |  |  |  |  |
| **Ground meats** | 33 | 6 | 1.0 | 19 | .77 | 4 | 1.0 | 24 | 2 | 1.0 | 12 | .51 | 1 | 1.0 |
| **Home chicken** | 30 | 1 | .01 | 15 | .06 | 0 | .003 | 28 | 0 | .07 | 14 | .30 | 0 | .17 |
| **Frozen chicken** | 14 | 1 | .67 | 4 | .01 | 0 | .18 | 11 | 0 | .57 | 4 | .18 | 0 | 1.0 |
| **Restaurant chicken** | 19 | 2 | .70 | 10 | .3 | 2 | 1.0 | 15 | 0 | .54 | 6 | .13 | 0 | 1.0 |
| **Raw milk** | 4 | 1 | .44 | 3 | .63 | 0 | 1.0 | 2 | 0 | 1.0 | 2 | .49 | 0 | 1.0 |
| **Water at home** |  |  |  |  |  |  |  |  |  |  |  |  |  |  |
| **Well** | 12 | 1 | .48 | 10 | .055 | 1 | 1.0 | 9 | 0 | 1.0 | 7 | .15 | 0 | 1.0 |
| **Others** | 49 | 8 |  | 25 |  | 5 |  | 39 | 4 |  | 18 |  | 2 |  |
| **Season** |  |  |  |  |  |  |  |  |  |  |  |  |  |  |
| **Summer** | 42 | 7 | .13 | 23 | .20 | 5 | .045 | 23 | 2 | 1.0 | 11 | 1.0 | 1 | .42 |
| **Winter** | 13 | 5 |  | 10 |  | 5 |  | 7 | 1 |  | 4 |  | 1 |  |

^α^ The cases with fluoroquinolone (ciprofloxacin) resistant *C. jejuni* infections.

^β^ The cases with tetracycline resistant *C. jejuni* infections.

^γ^ The cases with fluoroquinolone-tetracycline resistant *C. jejuni* infections.

***^δ^***  From χ^2^ test or Fisher's exact test

† The counts for animal contact and food consumption were not mutually exclusive for each category, thus *p*-value for each category was calculated.
