# Supplementary material for: Antimicrobial Susceptibility Profiles of Human Campylobacter jejuni Isolates and Association with Phylogenetic Lineages
Source: Front Microbiol. 2016 Apr 26;7:589. doi: 10.3389/fmicb.2016.00589 (PMC4845714; doi:10.3389/fmicb.2016.00589)
Supplement: Supplementary file 2 [file Table_2.DOCX]

**Supplementary Table 2.** **Mutations in the 23S rRNA genes from Multiple Drug Resistant *C. jejuni* isolates**

| **Strain** | **ST** | **MIC (μg/ml) for Azithromycin** | **Resistance profile** | **Nucleotide mutation relative to**  ***C. jejuni* ATCC33560 23S rRNA** | | | | |
| --- | --- | --- | --- | --- | --- | --- | --- | --- |
|  |  |  |  | **A2074T** | **C2097T** | **C2113T** | **T2172G** | **T2252C** |
| TW16607 | 6 | ≥64 | AziCipEryNalTel | NS |  | S | S |  |
| TW16657 | 5221 | ≥64 | AziCipEryNalTel | NS | S | S | S |  |
| TW16735 | 7010 | 0.12 | CipNalTet |  |  | S | S | S |

S; synonymous mutation, NS; non-synonymous mutation
