# Supplementary material for: Antimicrobial Susceptibility Profiles of Human Campylobacter jejuni Isolates and Association with Phylogenetic Lineages
Source: Front Microbiol. 2016 Apr 26;7:589. doi: 10.3389/fmicb.2016.00589 (PMC4845714; doi:10.3389/fmicb.2016.00589)
Supplement: Supplementary file 4 [file Image_2.PDF]

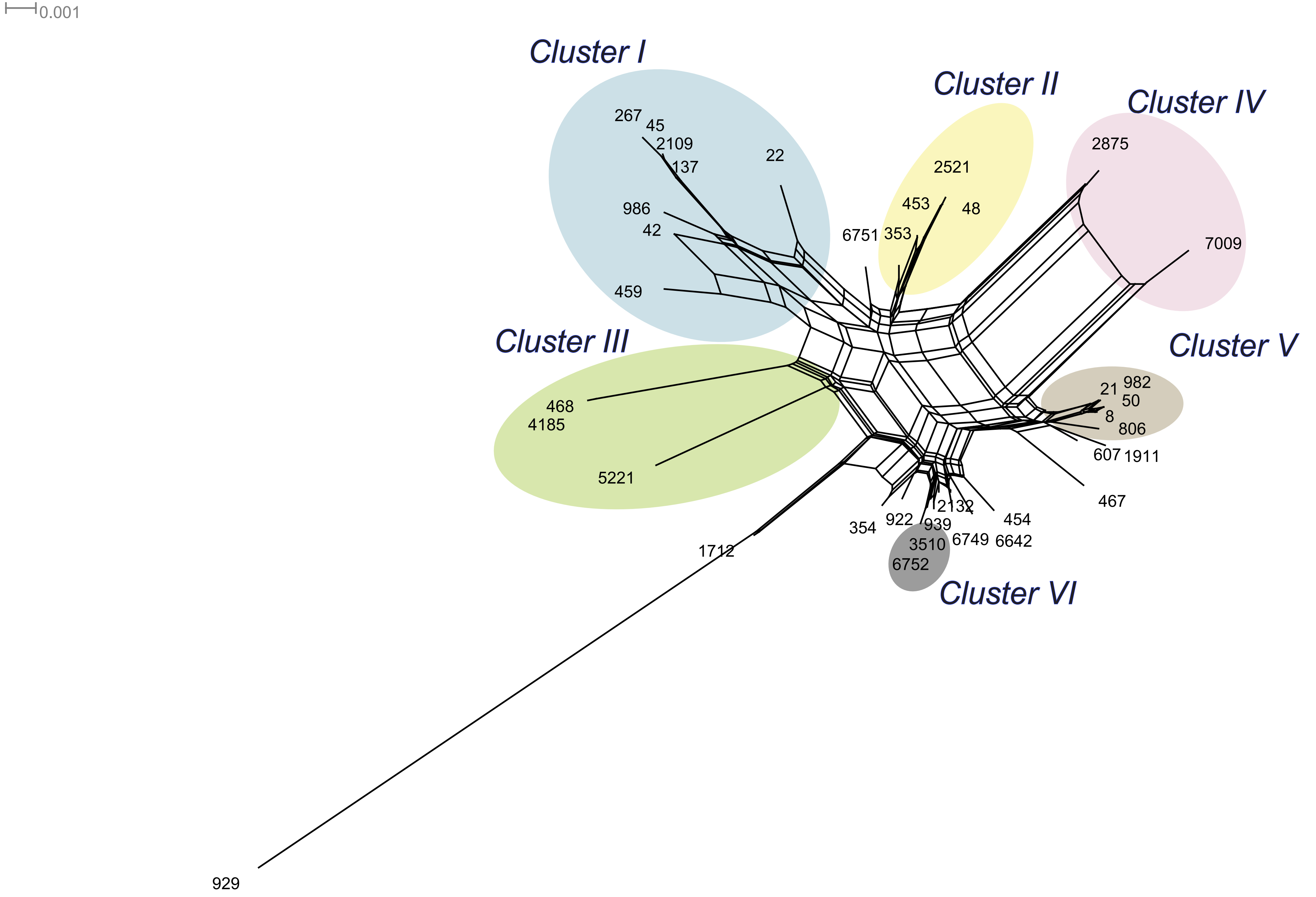

Supplementary Figure 2. Recombination among 36 sequence types (STs) from Michigan confirming the six clusters identified in the Neighbor-joining phylogeny.
