# Supplementary material for: Antimicrobial Susceptibility Profiles of Human Campylobacter jejuni Isolates and Association with Phylogenetic Lineages
Source: Front Microbiol. 2016 Apr 26;7:589. doi: 10.3389/fmicb.2016.00589 (PMC4845714; doi:10.3389/fmicb.2016.00589)
Supplement: Supplementary file 3 [file Image_1.PDF]

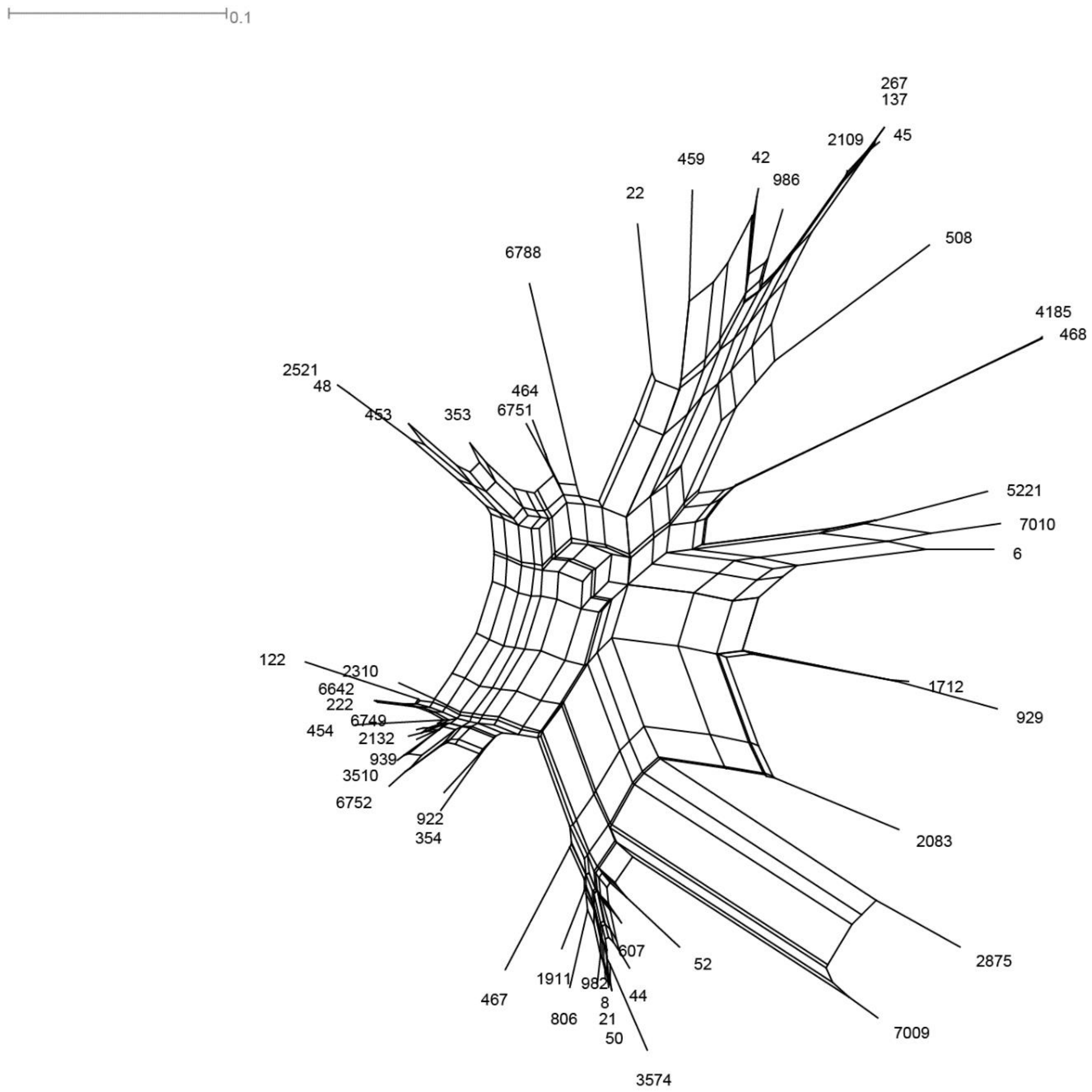

Supplementary Figure 1. Phylogenetic network highlighting recombination among sequence types (STs) from all isolates (*n*=94) based on the 144 parsimonious informative sites (PHI=0.0)
